# Ethical guideposts to clinical trials in oncology

**DOI:** 10.3390/curroncol13020004

**Published:** 2006-04

**Authors:** M. Bernstein

**Affiliations:** * Division of Neurosurgery, Toronto Western Hospital, University of Toronto, Toronto, Ontario

**Keywords:** Bioethics, clinical trials, ethics, conflict of interest, informed consent

## Abstract

Clinical research with human subjects is an ethically challenging task requiring ultimate trust on the part of patients and ultimate respect for persons on the part of clinical researchers. The author provides a simple framework to help researchers ensure the ethical integrity of a clinical trial in oncology.

## 1. INTRODUCTION

In the last few decades, considerable effort has gone into improving the rigorous assessment of the scientific validity and integrity of the randomized clinical trial (rct) 1,2. These steps are based largely on the invaluable work of the Evidence-Based Medicine Working Group (ebmwg) 2. A strong initiative is also under way to standardize how the results of rcts are reported 3. However, a pronounced discordance in attention between the scientific and the ethical dimensions of clinical research is apparent and is reflected in the emphases of ebmwg and of consort (Consolidated Standards of Reporting Trials Group) 2,3. Ethical issues are nonetheless assuming greater prominence in the conduct, interpretation, and use of clinical research, particularly as researchers explore new frontiers that not only invite but command rigorous ethical scrutiny.

Although most clinical investigators are virtuous and well-meaning doctors, it is easy for one to unknowingly and unwittingly transgress ethical boundaries, just as it is easy for a clinical oncologist without proper training in clinical trial design to use improper methodology. Some ethical dimensions are obvious because of common sense, common practice, or common law—for example, the requirements to submit a rct to the relevant institutional research ethics board (reb) and to obtain informed consent from research participants. Others dimensions are subtle and nuanced—such as the non-financial conflicts of interest experienced by clinical investigators during the course of clinical research. Furthermore, what constitutes “clinical research” is not always clear. For example, clinical innovation hovers on the border between clinical research and the inevitable advancement of the art and science of clinical practice 4.

## 2. DISCUSSION

Most of the literature on the ethical conduct of research consists of commentary or theoretic analysis, although a small number of papers have attempted to formulate usable frameworks 5–7. This article offers a simple and practical framework that clinical investigators can use to help assess the bioethical integrity of a clinical trial in oncology (Table I). The framework is based on the ethical principles of fairness, respect for persons and autonomy, the available literature, and the author’s personal experience with rcts. All points are presented in the past tense, but it is implicit that the questions should be asked and answered before a clinical trial is launched.

### 2.1 Was REB Approval Obtained?

When a hypothesis is to be tested in a clinical trial, most clinical investigators know that submitting the protocol to the institutional reb is an absolute requirement. The job of the reb 8,9 is to make sure that the research is ethically sound and to suggest ways to improve it, such as simplifying the consent form. Most protocols are improved by reb scrutiny, but these ethics boards are often understaffed, overworked, and vulnerable to criticism over how well and quickly they fulfil their mandates. In fact, the problem is escalating because the volume of clinical research at hospitals is continually increasing. It is not unfair to say that anxiety is growing among many clinical investigators about how accurately and promptly their clinical protocol will be assessed by their reb.

#### Guidepost 1

All clinical trials should be submitted to the clinical investigator’s institutional reb. The investigator should be ready to wait as long as several months for a response and should be prepared to receive many suggestions, some of which will be considered inappropriate or misguided. Investigators should do their best to comply with all suggestions or to explain clearly why compliance is impossible. The language in the consent form should be simple, but the explanations should be thorough.

### 2.2 Did All Patents Give Fully Informed Consent?

Informed consent is arguably the most important ethical dimension of research on human subjects, and yet it is arguably the most difficult to truly achieve 10,11. Fully informed consent has three fundamental components 12:

- Adequate disclosure of information
- Full patient capacity to comprehend the information
- Voluntariness or freedom of the patient to make a decision

For a rct to be justified, a state of clinical uncertainty about the relative merits of a trial’s arms—that is, the groups or methods being compared—must exist. This requirement alone is a difficult concept for most patients to grasp, and it is a sufficiently nuanced and sophisticated concept that even researchers exhibit inconsistency in grasping this very fundamental premise behind clinical research 13. *A priori,* a patient is entirely unable to know in advance whether participation in a study might be of personal benefit. The clinical investigator must be completely honest about presenting the experimental nature of the treatment being offered and must avoid propagating the widespread therapeutic misconception in which the patient believes that an offer of an opportunity to access a beneficial therapy is being extended 14,15.

Furthermore, clinical investigators cannot possibly predict every foreseeable complication of an experimental therapy, because previously unknown and unencountered complications can arise in the course of clinical research. This was the case in the first rct of high-activity interstitial brachytherapy for *de novo* glioblastoma multiforme: two patients developed devastating strokes from radiation injury to the middle cerebral artery 16.

Full capacity is arguably impaired in most patients being confronted with the daunting task of trying to digest all the information concerning a complex trial and making a decision that may have an impact on their quality of life or very survival. Voluntariness may also be adversely affected by a myriad of forces. In the final analysis, the most important component in clinical decision-making and the consent process for many patients may simply be their trust in the clinical investigator 17.

#### Guidepost 2

Clinical investigators must do their honest best to explain everything a reasonable person would want or need to know before entering a clinical trial. They should be honest about the unknown benefits of the therapy being studied. Simple language should be used in conversation with patents, and the investigator must be prepared to have more than one meeting with the patient if necessary. The investigators must be ever vigilant in maintaining complete respect for research subjects and in ensuring that every subject’s autonomy and dignity are preserved at all times.

### 2.3 Were the Eligibility Criteria for Entry into the Study Fair and Appropriate?

To increase internal validity, inclusion and exclusion criteria for clinical trials are made relatively stringent. This stringency is important, because the expenditure of human and fiscal resources required to run a clinical trial must be justified by the strong likelihood that scientifically valid and usable information will result. The trade-off may be reduced applicability of the results—that is, the results may be applicable to only a small subset of patients with the disease under study.

Clinical investigators must make sure, as best as possible, that access to a clinical trial is equitable and fair to all potential study subjects. Some examples of unfair access are egregious—for example, exclusion of vulnerable populations based on the disease under study, race, or type of health insurance 18. More common and everyday examples are “near-misses,” when patients fall just short of attaining eligibility for a trial. For example, how might a 67-year-old woman with breast cancer feel if a new trial of chemotherapy were to exclude her because the criterion for age was 65 years and under?

#### Guidepost 3

Clinical trials must be designed to provide the best possible opportunity for a definitive result without making the inclusion criteria narrower than absolutely necessary. But clinical investigators must be sensitive to patients who feel disadvantaged by ineligibility for entry and must be prepared to provide information to mitigate their feeling of being “left out.”

### 2.4 Was the Harm:Benefit Ratio Favourable to the Patients Who Entered the Study?

Before any clinical trial, detailed analysis of the potential harms of the experimental arm in particular must be weighed, and all research in animal studies and in phase i and ii human studies must be considered. The evidence must point in the direction of the experimental therapy having a strong—or, at minimum, a reasonable—chance of providing therapeutic benefit to patients. The uniformly dismal prognosis of a disease (for example, recurrent glioblastoma multiforme) should not be used to justify a study of an experimental treatment with an unreasonably high risk.

Complications, quality of life, and survival in each arm must be monitored on an ongoing basis, with the proviso that the study can be stopped if complications are excessive or if the outcome in either arm appears incontrovertibly worse or better than in the other 19. An independent safety and monitoring committee, well-versed in the possible pitfalls of inaccurate assessment of results, particularly early in a trial, should oversee the rct 19,20.

#### Guidepost 4

No matter how exciting an experimental therapy may appear in animal or early clinical observations, clinical investigators must strongly consider all foreseeable harms and be watchful for unanticipated ones. An independent monitoring committee should be sought to oversee the rct, with the power to stop the trial under certain circumstances.

### 2.5 How Were Patients Who Demanded the Experimental Therapy Off-Study Handled?

As patients become more informed and self-advocating, many hear about an experimental therapy and feel strongly enough about it to demand the treatment without wishing to risk randomization that gives them only a 50% chance of receiving it. How should such situations be handled?

Little has been written on this difficult issue 21, but as a starting point, providing the new treatment on demand must be considered intrinsically unfair to those who agreed to randomization. It could even bias the study results, because unknown factors associated with more-vocal patients might correlate with better or worse outcomes. Anything that could bias the result of a rct must be considered inherently unacceptable, because bad science is bad ethics. If another centre is known to provide the experimental therapy off-study, the investigator should provide the patient with that information and assist with a referral for the therapy if the patient desires it.

#### Guidepost 5

When a patient demands an experimental therapy off-study, the clinical investigator should not comply. The investigator should explain the reasons to the patient and should provide any available information on securing the treatment elsewhere.

### 2.6 Was the Study Placebo-Controlled, and If So, Was the Placebo Ethical?

Much has been written about the ethics of placebos in clinical investigation 22–24. Obvious ethical issues aside, the well-recognized placebo effect may contaminate the scientific results of a clinical trial 25. Use of placebos may expose participants to levels of risk that are unacceptable even if informed consent has been given. Placebos also produce an inherent level of deception, in spite of informed consent having been obtained. Some authors argue that placebos are acceptable under certain circumstances 23; others feel that placebos are generally unethical 22,24.

Placebos in surgical oncology trials (that is, sham surgery) must be rare, but precedents exist for invasive placebo-controlled arms in other surgical trials. A recent example involved placement, on the placebo arm, of an intraventricular catheter into the brains of patients with Parkinson disease 26. A recent survey of clinical researchers overwhelmingly endorsed the scientific value of sham-surgery controls, but only a minority of respondents (22%) believed that an invasive sham control is ethically justified 27. The use of placebos in medical oncology trials is common 28–30, and while not exposing subjects to risk, such a strategy exposes those patients to deception in spite of informed consent.

#### Guidepost 6

Any placebo in a rct that tips the harm:benefit ratio unfavourably should be considered unethical. This conclusion would obtain for all imaginable surgical placebos and perhaps for fewer medical placebos. Benefit that accrues to the scientific validity of a study cannot come at any foreseeable—and therefore avoidable—risk to participating patients.

### 2.7 Was the Effect Being Sought Clinically Meaningful or Just Statistically Significant?

Clinically relevant outcomes are a desirable subset of study outcomes that are also statistically significant. Traditionally, power calculations have been derived on statistical grounds; they must be rigorously performed to avoid the waste of human and fiscal resources associated with underpowered studies 31. Literature on minimally important clinical differences, which patients find relevant when asked, is also emerging 32. Indications are that a mismatch may exist between patients’ expectations of treatment and actual outcomes from clinical interventions 33. A null study provides an ethically acceptable result because it may obviate unnecessary human costs (that is, complications) and fiscal costs by recommending discontinuation of a fruitless experimental therapy. This was the case with interstitial brachytherapy for malignant gliomas mentioned earlier 34.

Besides quantitative outcomes such as prolongation of survival, time to progression, and 2-year survival (for example), qualitative outcomes such as quality of life are assuming greater importance in clinical trials, especially in oncology 35–37. Many would argue that no rct should be conceived or executed without measurement of quality of life as a primary outcome measure.

#### Guidepost 7

Power calculations for a rct must be rigorously performed, and a trial should be designed to discover differences that are clinically relevant to patients. If such information is unavailable, the clinical investigator should try to estimate what reasonable people would consider a worthwhile study outcome. Quality-of-life measurement should be built into every rct in oncology.

### 2.8 Were There Any Conflicts of Interest for the Study Investigators?

Financial conflicts of interest (cois) at an individual or institutional level have obvious potential as sources of bias or coercion that can influence rcts. Because such conflicts are becoming well-recognized, nowadays all such potential cois have to be transparently communicated to peers 38,39. But should patients be made aware of cois? Most investigators avoid confronting this issue. Yet a financial coi could affect a clinical investigator’s aggressiveness in recruiting patients to a rct (for example), and in the extreme scenario, could alter judgments about who is an appropriate candidate for a study.

Non-financial cois may be quite subtle and may exist for many reasons. For example, secret belief by the clinical investigator that the experimental therapy works can contribute to a more aggressive stance in recruiting patients, out of a desire to help them. Alternatively or in tandem, aggressive recruitment translates into quicker completion and earlier publication of the study, with resultant career advancement.

Clinical investigators must become aware of such influences. Some purists believe that the clinical investigator in a rct should not be the clinician actually caring for the patient 40. Others believe that, with vigilance to avoid subtle coercion or taking advantage of patients’ profound trust, care by the investigator is ethically acceptable 41. The situation of care by the investigator is likely necessary, because it is clinicians interested in exploring new therapies for their patients who generate local interest in the conduct of clinical trials. Without the integral involvement of these individuals, the clinical research study would likely not be initiated or attain success.

#### Guidepost 8

Clinical investigators must be aware of financial and non-financial forces that, through their powerful influence, can produce tension between the role of investigator and the role of healer. Clinical investigators must actively resist any temptation to allow these forces to unduly influence their behaviour in the investigator role. Every financial coi must be disclosed.

### 2.9 Were Patients Who Entered the Study Paid, and If So, Is This Ethical?

Although reimbursement is a relatively uncommon practice in oncology rcts, participants in some studies are reimbursed at minimum for expenses such as travel and parking associated with the extra clinic visits entailed by the trial. In some trials, however, participants are reimbursed above and beyond the real costs accrued 42,43. Any payment can be seen as a form of enticement or undue influence for a prospective subject to join a rct and therefore must be considered an ethically undesirable practice 44. Payment negatively influences the voluntariness component of informed consent discussed earlier.

#### Guidepost 9

Clinical investigators should not allow research subjects to be paid for participation in a rct.

### 2.10 Did All Co-authors’ Contributions Justify Authorship in the Study Publication?

Fabricated data, spurious authorship, and duplicate publication continue to be unethical practices in the publication of biomedical research 45,46. The only exception for multiple identical publications is position papers such as the statement on clinical trial registration by De Angelis and colleagues 47, which appears in about thirty different journals. And many would question the ethics even of this practice.

Currently, written justification of author contributions is required by many peer-reviewed journals. Every author listed must have earned credit for the manuscript by developing the study concept and design, by generating data, by writing the paper, by doing substantive editorial work, or by undertaking some combination of these components. Individuals who have made meaningful contributions must not be omitted. Attention must also be given to the order of authorship, which should be fairly assigned by equitable consensus among the co-authors, according to their relative contributions.

#### Guidepost 10

Clinical investigators must make sure that authorship of the results of a trial honestly and fairly reflects the work of all who contributed and only of those who did. Journals should probably request justification of authorship.

## 3. CONCLUSION

A simple framework that addresses essential elements of the bioethical integrity of a clinical trial in oncology has been presented here. It would be presumptuous to suggest that this framework is exhaustive or authoritative, but perhaps it can act as a springboard for investigators considering the ethical dimensions of their work. Also, this framework is subjective and largely reflects the beliefs of the author. No class i evidence is available to inform us about the issues raised—just attention to basic ethical principles and theories and thoughts from other thinkers in these areas.

Good clinical research is ultimately ethically desirable because it represents an attempt by investigators always to strive to improve outcomes for their patients. However, good intentions are not sufficient. Good bioethical conduct of clinical research is not widely taught in our undergraduate or graduate curricula—and even if it were, the face of research is changing so rapidly that new ethical dilemmas arise continually to challenge clinical investigators.

## Figures and Tables

**Table I:** Guide to an ethically sound clinical trial

Was approval obtained from the research ethics board? Did all patients give fully informed consent? Were eligibility criteria for entry into the study fair and appropriate? Was the harm:benefit ratio favourable to the patients who entered the study? How were patients who demanded the experimental therapy off-study handled? Was the study placebo-controlled, and if so, was the placebo ethical? Was the effect sought clinically meaningful or just statistically significant? Were there any conflicts of interest for the study investigators? Were patients who entered the study paid, and if so, is this ethical? Did all contributions from co-author justify authorship of the study publication?
